# Schooling upheaval during COVID-19: troubling consequences for students’ return to school

**DOI:** 10.1007/s13384-022-00572-x

**Published:** 2022-09-21

**Authors:** Leanne Fray, Felicia Jaremus, Jennifer Gore, Jess Harris

**Affiliations:** 1 CT Building, The University of Newcastle, University Drive, Callaghan, NSW 2308 Australia; 2 Teachers and Teaching Research Centre, School of Education, University of Newcastle, Newcastle, Australia

**Keywords:** Student well-being, COVID-19, Pandemic, Public school, Primary education

## Abstract

Efforts to contain the COVID-19 virus resulted in various stay-at-home orders and school closures around the globe, causing unprecedented disruption to the lives of children and generating grave concern for their well-being. This study draws on phone interviews with 12 teachers and 6 school leaders from 13 government schools in New South Wales, Australia, to provide insight into how students fared on their return to school after the first wave of COVID-19 in 2020. The interviews highlighted negative consequences for many students including increased stress and anxiety and decreased engagement. This evidence suggests that even a comparatively short period of school closure can drive troubling changes in students’ well-being and behaviour following their return to school. Given far more challenging conditions arising from the pandemic, both elsewhere in Australia and globally, we argue that attending to student well-being is as important as ensuring academic achievement and must be a key focus of policy makers and education systems moving forward.

## Introduction

The COVID-19 pandemic has disrupted schooling and the lives of students on an unprecedented scale. To help contain the virus, governments around the world have periodically closed entire schooling systems, ordering parents to keep their children home, where possible, during periods of high virus transmission. These mandates have affected more than 90% of the global student population, disrupting the lives of more than 1.5 billion students in 2020 alone (Psacharopoulos et al., 2020; United Nations, 2020b). While many education systems have been able to support learning from home during lockdown periods, with teachers rapidly moving to online and distance delivery of lessons when needed, students have nonetheless been physically isolated from their peers and teachers. Many have also been exposed to heightened levels of health and economic unease through the media and in their homes during this collectively traumatising event (Fitzgerald et al., 2021).

This situation has raised concerns, internationally, about the impact of COVID-19 on student well-being (Otu et al., 2020) and a possible future pandemic ‘wave’ of youth mental health crises (Grubic et al., 2020). Urgent calls for research on the impact of COVID-19 on student well-being have been issued to clarify the magnitude of, and mitigate, these global mental health challenges (Grubic et al., 2020; Otu et al., 2020). We contribute to the development of such a body of evidence via interviews with primary school teachers and school leaders about the effects of COVID-19 on their students’ well-being. Interviews were conducted during the post-lockdown return to school, following the 2020 pandemic wave in New South Wales (NSW), Australia.

### The Australian COVID-19 context

Compared to many locations in the world, Australia has been relatively unscathed by COVID-19. While the situation is evolving and new risks emerge, the number of cases in Australia was comparatively low prior to mass vaccination (in December 2021, at the time of writing, 219,118 cases and 2,056 deaths in a population of nearly 26 million). As a result, students in NSW experienced comparatively shorter lockdowns than those internationally, particularly during the first year of the pandemic (Patrinos et al., 2022).

During 2020, when this study took place, most Australians experienced a single nation-wide lockdown for eight to ten weeks, beginning in late March, which instructed people to stay home but kept schools open *only* for children of ‘essential workers’. In NSW, Australia’s most populous state, the lockdown was lifted after the first wave of cases was under control in late May and early June. During 2021, localised lockdowns continued, particularly in the capital cities of Melbourne, Sydney, Brisbane, and surrounding areas. The Delta variant of COVID-19 emerged in Australia in mid-2021 and large proportions of the south-eastern states of Australia were forced into lockdown, with students in NSW once again learning from home, this time for up to 14 weeks. While the impact of new variants, such as Omicron which appeared in late 2021, is still to be determined, there have been no additional lockdowns since mass vaccination in late 2021. During 2022, new ways of managing the pandemic emerged. Students and teachers testing positive to COVID were required to spend seven days in isolation and schools remained open. Nevertheless, understanding how students fared during the first period of school closure has relevance for subsequent disruptions to normal schooling.

It must be noted that the first signs of the 2020 pandemic emerged during Australia’s ‘Black Summer’, following years of prolonged drought. Record fires devastated Australia’s natural environment, impacted communities, and lowered air quality in the final months of 2019 and early months of 2020 (Berger & Reupert, 2020). The ‘Black Summer’ bushfires were responsible for the direct deaths of 33 people and the indirect deaths of more than 450 people who died due to smoke inhalation (Bradstock et al., 2021; Cook et al., 2021; Hitch, 2020). With more than a billion animals killed and 18.6 million hectares of land burnt, the Australian public, including school aged-children, entered the COVID-19 pandemic with significantly increased rates of depression and concern about the future (Arjmand et al., 2021). The closure of schools, with its loss of connection, emotional support, and stability for children and families (Brown et al., 2020) was, therefore, part of a cumulatively stressful period for the nation.

### Student well-being in Australia during COVID-19

It is well-established that, across the entire Australian population, well-being declined and anxiety increased significantly during the 2020 lockdown (Arjmand et al., 2021). Such effects were not limited to adults. Australia’s only national youth helpline (Kids Helpline) saw a monthly increase of 9.9% in calls from young children (aged 5–12) between February and August 2020, with callers expressing concerns about mental health, family relationships, suicide, and self-harm (Batchelor et al., 2021). Similarly, a survey of 760 Australian adolescents (aged 12–18) found three quarters of the sample had worsening mental health, with just under half (48.3%) reporting distress levels indicative of mental illness (Li et al., 2021).

These negative impacts were observed by teachers in their online engagements with students while learning from home, with some reporting they focussed, out of perceived necessity, on students’ well-being rather than curriculum content (Forster, 2020). Such concern was especially great for vulnerable students who rely on school as a place of safety and/or may be too young to comprehend sudden changes in society (Brown et al., 2020; Drane et al., 2020; Flack et al., 2020). Certainly, vulnerable students were seen to be at greater risk of psychosocial challenges, technological barriers to learning, and school disengagement, which could result in persistent disadvantage over the long term (Brown et al., 2020; Drane et al., 2020).

We note, however, that such concerns have mainly been raised in reports (Brown et al., 2020; Flack et al., 2020), media articles (Forster, 2020), and reviews of prior literature (Drane et al., 2020), with little peer-reviewed empirical research available on student well-being during COVID-19. Instead, speculation, projection, and moral panic about the long-term consequences for both learning and well-being have been rife (Borko et al., 2010; Dodd et al., 2020; Fuchs-Schundeln et al., 2020; Haiek, 2020; Posso, 2020). Without directly relevant empirical studies, most concerns have been extrapolated from studies about the impact of natural disasters and other crises (such as school shootings) which demonstrate that any period of upheaval involving limited social contact can be a source of trauma for students and may result in limited engagement with schooling (Mohay & Forbes, 2009). Our own research (Gore et al., 2021) and the national testing scheme (ACARA, 2021) indicate that student *learning* in NSW primary schools during the same period did not suffer significantly. Academic achievement was consistent with pre-pandemic levels. However, in what ways was student *well-being* affected by the 2020 school closure? To what extent are current concerns warranted?

To address these questions, we investigated the impact of COVID-19 on students, asking teachers and school leaders first to reflect on their own experiences and the experiences of their students during the learning from home period and then to share their perceptions of students when they returned to school.

## Methodology

We did not set out to study the effects of COVID-19. In fact, when the first wave of the pandemic struck, we were in the middle of conducting a mixed methods randomised controlled trial (RCT) examining the effects of Quality Teaching Rounds professional development on student achievement and the quality of teaching, which had to be postponed (see Taggart et al., under review for an account on the consequences of this decision). Rather than waste the baseline data collected in early 2020 (prior to the pandemic), teachers and school leaders in participating schools (*n* = 51) were invited to take part in an alternative project examining the effects of COVID-19 on student achievement (Gore et. al., 2021), teacher morale and efficacy (Fray et al., 2022), and student well-being.

This paper addresses the impact on student well-being after schools closed for a period of eight to ten weeks during Australia’s national lockdown in 2020. Well-being is a multi-dimensional concept (Kellock, 2020; Main, 2014) that takes into account physical, mental, emotional, and social aspects of health as well as individuals’ capacity to achieve balance between facing challenges and having the resources needed to meet those challenges (Dodge et al., 2012). We chose not to impose any definition of well-being on interviewees, instead leaving the concept open to their interpretation.

Given limited research on student experiences during COVID-19 in Australia, we opted for an exploratory descriptive approach (Hunter et al., 2019), combining qualitative descriptive research designed to understand the “who, what and where of events” (Sandelowski, 2000, p. 339) with qualitative exploratory research designed to explore a poorly understood or under-researched phenomenon (Stebbins, 2012)—effects of COVID-19 on student well-being.

A sub-sample of teachers (*n* = 12) and school leaders (*n* = 6) from NSW government primary (elementary) schools (*n* = 13) was invited to participate in telephone interviews during September and October 2020 (Term 3 of a four-term school year). In Australia, the school year commences in late January and concludes in mid-December.

Teachers in this study were working with Year 3 and 4 students (aged 8–10 years) while school leaders brought a school wide perspective from their entire student cohort (Kindergarten to Year 6, students aged 5–12 years). Teachers have long been valued as informants on the experiences of students in general (see for example De Groot, 2010; Wingenfeld et al., 1998) and, more recently, on the specific issue of student well-being (Anderson & Graham, 2016; Graham et al., 2016). While we acknowledge the importance of hearing from children and young people directly, we decided not to speak with students given their young age (8–10 years) and the risk of exacerbating any feelings of anxiety. Moreover, it is typically the more confident, articulate students who volunteer their voices in primary school research settings (Mayes et al., 2019), hence the experiences of more vulnerable students can be overlooked.

Interviewees were asked questions about their experience during lockdown, the impact of lockdown on student well-being and achievement, challenges faced by students and their families, any known advantages to the ‘learning from home’ arrangements, and their perceptions of student well-being once school resumed full-time (see Appendix for a full list of interview questions). All interviews lasted between 30 min and one hour.

While we make no claims to the generalisability of results, the 13 schools involved in this study were selected to be broadly representative of the schools in the larger study, based on school location and socio-educational advantage (ICSEA).^1^ The sample demographics displayed in Tables 1 and 2 highlight the diversity of our sample. The schools were in inner regional (*n* = 4) and outer regional (*n* = 3) areas and in major cities (*n* = 6) (Table 1).

**Table 1:** Location of 2020 interview schools and participants

	Major city	Inner regional	Outer regional	Total
Schools	6	4	3	13
School leaders	3	2	1	6
Teachers	5	4	3	12

**Table 2:** Sociodemographic characteristics of schools in interview sample

	ICSEA	Language background other than English (%)	Aboriginal or Torres Strait Islander students (%)	Number of teachers (2019)	2019 enrolment
School 1	Low	< 10	61–70	31–40	451–500
School 2	Low	< 10	31–40	< 10	51–100
School 3	Low	< 10	41–50	11–20	251–300
School 4	Low	< 10	< 10	< 10	< 50
School 5	Low	91–100	< 10	21–30	301–350
School 6	Mid	11–20	11–20	21–30	451–500
School 7	Mid	< 10	< 10	11–20	251–300
School 8	Mid	< 10	< 10	< 10	151–200
School 9	Mid	71–80	< 10	11–20	251–300
School 10	Mid	< 10	< 10	11–20	301–350
School 11	Mid	< 10	< 10	21–30	351–400
School 12	Mid	11–20	< 10	11–20	251–300
School 13	High	71–80	< 10	41–50	751–800

In order to protect the anonymity of schools, ICSEA is reported as low (< 950), mid (ICSEA 9501–1050), and high (ICSEA > 1050) and all other variables are reported as a range

The school Index of Community Socio-Educational Advantage (ICSEA) ranged from 810 (least advantaged school) to 1141 (most advantaged). The percentage of students with a language background other than English (LBOTE) ranged from 0% (Schools 2 and 4) to 98% (School 5). Aboriginal and Torres Strait Islander student enrolment ranged from 1% in School 5 to 61% in School 1. The number of classroom teachers in schools ranged from 3 (School 4) to 41 (School 13) and student enrolments ranged from 29 (School 4) to 777 (School 13) (Table 2).

### Analysis

All interviews were audio-recorded, professionally transcribed, and read by at least two members of the research team. Coding of the interview data was undertaken using the NVivo™ software program (QSR International, 2020) to identify the “essential elements of the research story” (Saldaña, 2016, p. 9). The first wave of coding generated 26 nodes while discussion with all researchers reduced the number to 22. The researchers then met to discuss and compare codes to ensure consistency and consensus (Harry et al., 2005).

In line with our research design, the coded insights of teachers and school leaders were then thematically analysed. This analysis generated the two key themes of ‘unprecedented upheaval to schooling’ and ‘worrying impact on student well-being’. In reporting the substance of the interviews, pseudonyms are used to protect the anonymity of participants and schools involved in the study.

## Results

### Unprecedented upheaval to schooling

Almost overnight, school children in NSW were buffeted by the rapid and unexpected move to new forms of learning, high levels of family and community angst, the loss of key rituals and celebrations in school and at home, limited connections with peers and, upon return to school, a reduced curriculum.

The rapid move to learning at home caused significant stress, frustration, and anxiety for families, as one teacher explains:

I spoke to some parents who were really stressed out—obviously by COVID itself, by work, people that were working from home, people that had no work, people that really... I have one dad who was telling me, “I feel like a failure. I can’t help them. I’ve got no idea what they’re doing. I can’t make them do anything”. So, I really started to find there became a point where the whole home learning itself was just really creating stress and some families were not able to engage in it at all. (Samantha, teacher, school 1, outer-regional, low ICSEA)

The unanticipated move to relying on new forms of technology caused further discontent for some students, and a sense of failure and disempowerment for many parents. This was particularly the case in regional areas, where access to the Internet was an ongoing challenge. Jessica, who teaches in a low ICSEA school, captures the multiple frustrations experienced by her students:

The problem is some of the kids got frustrated. …[For example, Jack] was getting frustrated because his mum wouldn’t log in and help him and she said, “Oh, I can’t do this”… Then we had other [students] who couldn’t [connect online] because they were out on farms and the Internet connection they had was so poor, it would drop out… and then they’d be frustrated because they’d spend the rest of their time trying to get back in and they couldn’t. …So, it was a bit of a debacle. (Jessica, teacher, school 1, outer regional, low ICSEA)

As Jessica﻿ points out, learning from home added multiple layers of frustration—student frustration with parents, parent frustration with technology, and shared frustration with schooling at home.

Other teachers, such as Kelly, highlighted the disruption to schooling-as-usual caused by placing key rituals and celebrations on indefinite hold, even when students returned to school.

[In] Kindergarten^2^ almost everything they do is singing… So yeah, no, we can’t sing or anything. We haven’t been able to go on excursions. So, camps, everything. The sport and the camps … especially for Year 6 has been really hard because that’s the carrot that you dangle around behaviour, and they’ve got nothing… [And then there’s] transition. So, our Year 6s—doesn’t seem like they’ll get their transition days to high school. But the same, we can’t run our Kindy transition. We usually have the kids on site over three weeks, different days and we can’t do that. There’s a whole massive, there’s just going to be like a whole big wave that’s going to affect all [of] next year’s [students] as well as this current year. (Kelly, teacher, school 10, inner regional, mid ICSEA)

Kelly is clearly concerned that the loss of those activities associated with major transition points in schooling (such as entering Kindergarten, leaving Year 6, entering high school) will have lasting negative effects on students.

While such curricular curtailment may help students catch up in specific key learning areas, such as the high stakes numeracy and literacy, the lack of extracurricular activities led to what one school leader described as ‘*sort of like Groundhog Day, day-in, day-out*’ (Rachel, school leader, school 13, major city, high ICSEA):

I’ve mentioned previously, we had a lot of extracurricular programs—they’ve all been stopped. So there’s nothing for the kids to look forward to. …Our [student] school leaders have had no reason to be publicly speaking or no reason to hold any events because you’re not allowed to hold any events. Teachers not being able to have their buddy classes going because you can’t mix stages [year groups]. So, there’s not much for the kids to look forward to. (Kylie, school leader, school 9, major city, mid-ICSEA)

As a school leader with responsibility for ensuring the well-being of students in her school, Kylie paints a bleak picture associated with shutting down extracurricular life—leaving students with little *to look forward to*.

Although such circumstances were reported by participants from all schools, the upheaval to schooling varied based on the specific location and student population. School leader Rachel points to the vexing impact of anti-Chinese racism in Australia, fuelled by political commentary, such as that provided by US President Trump, who repeatedly called COVID-19 the “Chinese virus” (Tan, 2020; Viala-Gaudefroy & Lindaman, 2020; Walden & Yang, 2020). The impact on students in communities with a high proportion of residents from Chinese backgrounds was amplified when residents themselves were viewed as potentially dangerous in transmitting the virus, because of their heritage:

There was an enormous amount of angst here at the very beginning…both within our Chinese community and our non-Chinese community, that we might have been an at-risk school [of spreading the virus]…I think one of the primary things we had to do was make sure that *that* didn’t take off and have a life of its own. And we were very, very concerned about the impact on the welfare and well-being of our families, that there may have been some… racism is probably not the right word, but some over-concern, some misguided concern. (Rachel, school leader, school 13, major city, high ICSEA)

At the same time as schools like Rachel’s grappled with the destabilising effects of anti-Chinese sentiment, others in regional areas where there were no reported COVID-19 cases, felt relatively safe. In some instances, students were back at school long before the formal end to the school lockdown period:

We had a lot of families just go, “I’m sending my kids” and I think it was due to our geographic location of going, “there’s no COVID around, I feel safe, they’re going to school.” So, sort of from I think it was about Week 2 in Term 2 when we were sort of open for essential workers, the moment that that occurred we had 75% of our kids on site at least four days a week. (James, school leader, school 3, outer regional, low ICSEA)

While there is little doubt that the pandemic caused considerable upheaval to schooling, the commentary provided by Rachel and James indicates how factors such as race and geography contributed to greatly varying experiences for students during the first wave of the COVID-19 pandemic in NSW.

In sum, the first round of school closures in 2020 caused a major upheaval to schooling which included rapid adaptation to new forms of learning, loss of key milestones, and once they returned to school, a significantly reduced curriculum. Next, we turn to how teachers and school leaders understood the impact of these changes on student well-being.

### Worrying impact on student well-being

Almost all teachers and school leaders in our sample had significant concerns for the well-being of students after they returned to school, manifest in increased anxiety and aggression in the classroom, poor social interaction, decreased engagement, and a range of day-to-day behavioural issues. One school leader indicated she was seeing *“trends around student stress”* (Rachel, school leader, school 13, major city, high ICSEA), another reported *“more aggressive behaviours”* (Kylie, school leader, school 9, major city, mid-ICSEA), and a third observed “*self-harm and threatening self-harm*” among her student population (Lauren, school leader, school 6, major city, mid-ICSEA). Here, Chris articulates how COVID-19 seemed to compound pre-existing behavioural issues:

I had a lot more behaviour issues creeping up, and I’ve got students with anxiety. I’ve got students that are medicated. I’ve got students with learning needs…And I think all of it’s just being genuinely compounded by the stresses and pressures and the general not knowing what it’s going to look like… I would say, there’s a lot more anxiety. There were students [who are now] probably a bit less confident, which is probably a result of anxiety. I think that there was just generally worse behaviour when they came back. (Chris, school leader, school 11, major city, high ICSEA)

Teachers also reported poor social interaction among students on return to school, which they attributed to the lack of direct contact with peers during lockdown. One teacher went so far as to say that students “*couldn’t talk or interact with their peers anymore, like they’d lost it*” (Mateo, teacher, school 13, major city, high ICSEA). Samantha, a classroom teacher from a low ICSEA school, concurred:

I think it has really impacted them socially more than anything, and how they are engaging and relating to others. I feel that… the students are getting quite fatigued… they seem to be a bit more tired, not engaging as much, lots of behaviour issues, and stuff like that. (Samantha, teacher, school 1, regional, low ICSEA)

While great concern has been expressed about the effect of lockdowns on student academic achievement, this teacher commentary highlights the less publicised impact of a lack of social interaction, resulting in high levels of fatigue, increases in challenging behaviour, and a lowered capacity to engage in learning once students returned to the classroom.

Teachers also shared ongoing concerns about students’ engagement in learning. Those who had struggled to engage during the 8-week school closure period continued to have difficulties when they returned to school. School leaders, such as Lauren, linked this lack of engagement with deteriorating *mental well-being*:

So, students who didn’t engage during distance learning, have continued with a high level of difficulty re-engaging back in the classroom. So, yes, my staff has seen that COVID-19 has had a major impact on the mental well-being of the students who feel that they’re unable to keep up. … They just don’t try anymore. (Lauren, school leader, school 6, major city, mid ICSEA

Teachers, such as Mateo, concurred:

They weren’t mentally prepared to come to school…we had to deal with a lot of silly, silly, silly issues. For example, there might be someone biting someone, or someone saying something, like those issues that you would never expect, not from someone that’s nine or 10 years of age. Again, it was really over nothing…there wasn’t really any reason for them to be partaking in those activities or certain issues. Yeah, it was definitely a big, big change. (Mateo, teacher, school 13, major city, high ICSEA)

Such commentary highlights how unsettled students were as they returned to school.

Yet, despite consistent accounts of *“diminished well-being”* across schools as children returned to classrooms (Kylie, school leader, school 9, major city, mid-ICSEA), there was some variability. In particular, regional and remote areas appeared relatively protected by fewer COVID-19 cases in their communities. School leader, James, who was located in an outer regional community, testifies to the experience of students in rural communities:

I think probably the impacts for our students, I don’t believe they would have been as severe as someone from a city area as such. Like we had a lot of farming kids who were like ‘okay I’m just helping around the farm’… It was entirely okay, it’s a different learning yes, but they were connecting with their mum and dad, they were connecting with people that they don’t always get to connect with. (James, school leader, school 3, outer regional, low ICSEA)

In sum, while teachers overwhelmingly expressed concern about student well-being and reported increased stress and anxiety, poor social interaction, and decreased student engagement on return to school, it is worth noting that the impact on student well-being was not uniform. This is to be expected given differing circumstances in schools and families, differing challenges faced, and differing capacities for and ways of responding to the pressures.

## Discussion

Given international evidence that the well-being of young people and children is especially vulnerable to the effects of crises (OECD, 2020; United Nations, 2020a; Whaley et al., 2017), it is unsurprising that COVID-19 and the learning from home period generated grave concern about student well-being. Throughout the world, the long-term effects of the COVID-19 pandemic on students (UNESCO, 2020) remain largely unknown. Our teacher and school leader observations after just eight weeks of school closure are likely to depict the tip of the iceberg. Where schools closed for longer and/or restrictions were harsher, elsewhere in Australia and abroad, the effects on student well-being are likely to be more severe (Fontenelle-Tereshchuk, 2020; Forster, 2020; Lee, 2020; Maunula et al., 2021; Steinmayr et al., 2022; Zhang et al., 2020). The cumulative effects of repeated school closures, such as the additional 14 weeks associated with the Delta strain of COVID-19 in NSW in 2021 are yet to seen (Berger & Reupert, 2020; Brooks et al., 2020). Nonetheless, our evidence from 2020 indicates that, after a relatively short period of school closure, concern for student well-being in NSW schools is not only warranted but clearly an urgent matter for the broader educational community.

As we have documented, during the learning from home period, teachers and school leaders reported that student well-being was substantially impacted by high levels of anxiety in the broader community and within households (Fitzgerald et al., 2021; Lee, 2020). Many stories were told of families who struggled to support student learning, a challenge exacerbated by stress within the family home. Adapting to new ways of teaching and learning generated additional stress, particularly for families with limited digital literacy and/or access to the necessary technological resources to support learning from home (Rivero et al., 2022; Thorn & Vincent-Lancrin, 2021).

Given the concerns about school closures for student (Singh et al., 2020) and teacher well-being (Fray et al., 2022; Gore et al., 2020; Ziebell et al., 2020), as well as economic productivity (Joseph & Fahey, 2020), it is unsurprising that a widespread consensus emerged in favour of keeping lockdowns as short as possible. However, the reopening of schools, often framed as a return to normalcy and a time for helping students ‘catch up’ academically (Sonnemann & Goss, 2020), was far from normal.

A key message of this study is that the challenges experienced during the learning from home period were not simply alleviated once students returned to school. Rather, the cancellation of all extracurricular activities, and the resulting monotony of schooling, contributed to high levels of student fatigue and a rise in unacceptable behaviour, poor social interaction, and difficulties re-engaging in classroom learning. As Batchelor et al. (2021) argue, while the stress associated with families being confined to home for extended periods was likely to resolve when situational factors resolved, or when students returned to school, the effects on mental health may be observable over a much longer period.

Given that our teachers and school leaders were in primary schools, their reports of stress, anxiety, poor mental health, and even self-harm, when extrapolated to older student cohorts, signal potentially greater dangerous social and emotional effects on student well-being. Indeed, evidence from international studies consistently documents decreased well-being among adolescents (Engel de Abreu et al., 2021; Munasinghe et al., 2020). Clearly, significant investment in student well-being programs, for students in all years of schooling, will be essential to ameliorate long-term negative effects.

However, we argue, investment in student well-being programs needs to be appropriately targeted, rather than supporting broad, generic programs for all students. Teachers’ observations of aggressive behaviour and self-harm in some students demonstrate a need for urgent specialised help. In contrast, students whose well-being was minimally impacted are likely to require far less additional support.

### Limitations

Two key limitations of our study must be acknowledged. First, we are reporting on a single eight-week lockdown in one Australian state where the impact of the pandemic (overall) was much less severe than in other locations. Our participants came from widely diverse schools that are broadly representative of the schools in our larger sample. But we conducted a relatively small number of interviews (*n* = 18) in a state where there is a workforce of more than 30,000 primary school teachers. Even with this small sample, we have captured considerable diversity of experience and uncovered deeply worrying consequences of the school closure period.

Second, we did not speak to students themselves, instead relying on reports from teachers to understand how students fared once they returned to the classroom. While speaking to, or surveying, students would have provided firsthand accounts, we elected not to do so given the age of students (8–10 years old) and the added risk of exacerbating the considerable angst community members were already feeling due to conditions created by the pandemic.

## Conclusion

Given school closures in many parts of the world, concern for both student learning and student well-being has been profound throughout the COVID-19 pandemic. Substantial research has been conducted on student learning, with most studies reporting negative effects, particularly for younger children and those from low socio-economic backgrounds (Hammerstein et al., 2021; Patrinos et al., 2022). By contrast, studies conducted in NSW found the disruption to schooling during 2020 had minimal impact on students’ academic achievement (ACARA, 2021; Gore et al., 2021). Less research has addressed the effects on student well-being. The study reported in this paper demonstrates that even where student learning was not significantly affected, the upheaval to schooling caused by the pandemic is having worrying consequences for their well-being. Indeed, our analysis signals the likelihood of widespread, complex, and troubling effects on student well-being that we are only just beginning to understand.

Evidence is mounting that the COVID-19 pandemic continues to traumatise many Australian school students, with more than 50% of young people (aged 15–24) indicating the pandemic negatively affected their mental health (Mission Australia, 2021). We argue that urgent attention and vigilance are required, together with restorative forms of support, to help mitigate the impact of COVID-19 on student well-being. While it is important to ensure student learning is not compromised by school closures, it is equally vital that we invest in and address the less visible issues of mental health and well-being.

## Interview schedule

1. Can you tell me about the ‘learning from home’ arrangements implemented by your school this year?
2. How were you supported (by your school and/or the Department or others) in the transition to (and from) ‘learning from home’ arrangements?
3. What had been your experiences of using technologies to deliver lessons prior to the school shut down period?
4. What were the main challenges to you in delivering lessons via ‘learning from home’ arrangements?
  Possible prompt
5. To what extent do you think your experiences are representative of other teachers at this school?
6. Were there any advantages to you in delivering lessons via ‘learning from home’?
  *Possible prompts:*
7. What worked well?
8. Is there anything that you tried/used/implemented during ‘learning from home’ arrangements that you will continue to use in your school/teaching?
9. To what extent do you think your experiences are representative of other teachers at this school?
10. What challenges did you hear about from students and families while they were ‘learning from home’?
11. Do you know of any particular advantages for your students or their families in students learning from home? Any specific disadvantages?
12. In your opinion, what impact has COVID-19 had on student achievement? How was this monitored or assessed?
13. Has student engagement altered in anyway as a result of COVID-19? If so, how?
14. What effect (if any) has the break from traditional schooling had on student well-being?
15. Were there any differences in the ways you approached teaching your class once school resumed full-time? If so, how did your teaching differ after the lockdown period from before the lockdown period?
16. Anything else you would like to tell us about your experience this year?

## Footnotes

1 ICSEA is a standardised measure of school-level advantage in Australia. It has a mean of 1000 and a standard deviation of 100. It includes school location, parental education, and occupational level as well as the proportion of Indigenous students.

2 ^2^ Kindergarten is the first year of schooling in NSW primary (elementary) schools. Students are generally aged between 5 and 6 years.
